# Effects of Simulated Microgravity on the Physiology of *Stenotrophomonas maltophilia* and Multiomic Analysis

**DOI:** 10.3389/fmicb.2021.701265

**Published:** 2021-08-27

**Authors:** Xiaolei Su, Yinghua Guo, Tingzheng Fang, Xuege Jiang, Dapeng Wang, Diangeng Li, Po Bai, Bin Zhang, Junfeng Wang, Changting Liu

**Affiliations:** ^1^Medical School of Chinese PLA, Beijing, China; ^2^Department of Respiratory and Critical Care Medicine, The Second Medical Center and National Clinical Research Center for Geriatric Disease, Chinese PLA General Hospital, Beijing, China; ^3^College of Pulmonary and Critical Care Medicine, The Eighth Medical Center, Chinese PLA General Hospital, Beijing, China; ^4^Department of Academic Research, Beijing Chaoyang Hospital Affiliated to Capital Medical University, Beijing, China; ^5^Respiratory Diseases Department, PLA Rocket Force Characteristic Medical Center, Beijing, China; ^6^Department of Respiratory and Critical Care Medicine, Binzhou Medical University Hospital, Binzhou, China

**Keywords:** simulated microgravity, *Stenotrophomonas maltophilia*, physiology, biofilm, mobility, multiomic analysis

## Abstract

Many studies have shown that the space environment plays a pivotal role in changing the characteristics of conditional pathogens, especially their pathogenicity and virulence. However, *Stenotrophomonas maltophilia*, a type of conditional pathogen that has shown to a gradual increase in clinical morbidity in recent years, has rarely been reported for its impact in space. In this study, *S. maltophilia* was exposed to a simulated microgravity (SMG) environment in high-aspect ratio rotating-wall vessel bioreactors for 14days, while the control group was exposed to the same bioreactors in a normal gravity (NG) environment. Then, combined phenotypic, genomic, transcriptomic, and proteomic analyses were conducted to compare the influence of the SMG and NG on *S. maltophilia*. The results showed that *S. maltophilia* in simulated microgravity displayed an increased growth rate, enhanced biofilm formation ability, increased swimming motility, and metabolic alterations compared with those of *S. maltophilia* in normal gravity and the original strain of *S. maltophilia*. Clusters of Orthologous Groups (COG) annotation analysis indicated that the increased growth rate might be related to the upregulation of differentially expressed genes (DEGs) involved in energy metabolism and conversion, secondary metabolite biosynthesis, transport and catabolism, intracellular trafficking, secretion, and vesicular transport. Gene Ontology (GO) and Kyoto Encyclopedia of Genes and Genomes (KEGG) enrichment analyses showed that the increased motility might be associated the upregulation of differentially expressed proteins (DEPs) involved in locomotion, localization, biological adhesion, and binding, in accordance with the upregulated DEGs in cell motility according to COG classification, including pilP, pilM, flgE, flgG, and ronN. Additionally, the increased biofilm formation ability might be associated with the upregulation of DEPs involved in biofilm formation, the bacterial secretion system, biological adhesion, and cell adhesion, which were shown to be regulated by the differentially expressed genes (chpB, chpC, rpoN, pilA, pilG, pilH, and pilJ) through the integration of transcriptomic and proteomic analyses. These results suggested that simulated microgravity might increase the level of corresponding functional proteins by upregulating related genes to alter physiological characteristics and modulate growth rate, motility, biofilm formation, and metabolism. In conclusion, this study is the first general analysis of the phenotypic, genomic, transcriptomic, and proteomic changes in *S. maltophilia* under simulated microgravity and provides some suggestions for future studies of space microbiology.

## Introduction

In recent decades, with the development of medicine and space technology, microbial space safety has become one of the hot spots in modern medical research. The space environment involves microgravity, high pressure, low temperature, cosmic radiation, and malnourished environments (Horneck et al., 2010). However, microbes can still survive under such extreme conditions (Venkateswaran et al., 2014). Some studies have found microbes on surfaces, air filters and even in air samples from the International Space Station (ISS), and the diversity and abundance of microbes have been investigated (Checinska et al., 2015). *Staphylococcus*, *Enterococcus faecalis*, *Bacillus*, *Propionibacterium*, and *Corynebacterium* have been identified as the most common species. Studies have showed that the space environment has some impacts on the immune system and motor system of humans, as well as on microorganisms in astronauts, such as growth rate, drug susceptibility, carbon source utilization and chemical sensitivity, biofilm formation ability, and motility (Taylor, 2015; Nadeau et al., 2016; Vaishampayan and Grohmann, 2019; Zhang et al., 2019). However, the impact of the space environment on microbes varies on different bacteria. Assuring that astronauts are sound in body and mind and have a good working capability is challenging. During spaceflight, the spaceship provides insulation from cosmic radiation and extreme temperature to some extent. Therefore, microgravity is a key influencing factor on astronauts and microbes.

Given the constraint on spaceflight missions and the uncertainties in outer space experiments, land-based, high-aspect rotating vessels (HARVs) have been developed by NASA (Wolf et al., 1992). HARVs simulate microgravity by rotating cultures on an axis perpendicular to gravity, and the weight of microbes can be affected by low shear forces, which can suspend bacteria in a constant free fall state (Radtke and Herbst-Kralovetz, 2012). Over the decades, HARVs have been widely used to explore the physiological states of microorganisms in a simulated microgravity (SMG) environment (Nickerson et al., 2000; Wilson et al., 2002a,b; Lynch et al., 2004; Lawal et al., 2010; Castro et al., 2011; Rasamiravaka and El Jaziri, 2016). In addition, HARVs provide a first-rank suspension growth environment that imitates some of the aspects of microgravity, such as a reduction in fluid shear, minimal turbulent motion, and short sedimentation of bacteria or host cells (Hammond and Hammond, 2001). Some experts have verified the effectiveness of HARVs through mathematical analyses (Lynch et al., 2006). In addition, the reduction in shear stress within HARVs promotes the formation of 3D tissue aggregates (Sawyer et al., 2008).

*Stenotrophomonas maltophilia* (*S. maltophilia*), which was first isolated in 1943, is an emerging Gram-negative multidrug-resistant organism (MDRO) that is highly relevant to respiratory infections in humans (Hugh et al., 1963). *Stenotrophomonas maltophilia* is an important nosocomial pathogen that is closely related to infections, particularly in immunocompromised people. Early research has suggested that *S. maltophilia*-associated infections are more common in conjunction with severe pneumonia, chronic obstructive pulmonary disease (COPD), bacteremia, biliary sepsis, bone and joint infections, eye infections, endocarditis, and meningitis (Papadakis et al., 1995; Fujita et al., 1996; Landrum et al., 2005; Lai et al., 2006; Takigawa et al., 2008; Yemisen et al., 2008). Currently, research on *S. maltophilia* mainly focuses on its drug resistance and clinical anti-infection treatments (Sanchez et al., 2009; Amin and Waters, 2016). However, the impact of the space environment or SMG on *S. maltophilia* has not been thoroughly investigated, and few research has explored whether and how the space environment or SMG changes its growth rate, phenotypic characteristics, antibiotic susceptibility, carbon source utilization and chemical sensitivity, biofilm formation ability, and motility. In this study, we studied the phenotypic variations of *S. maltophilia* and used combined genomic, transcriptomic, and proteomic analyses to reveal the impact of SMG on *S. maltophilia*. Our research results may provide new ideas for the prevention and treatment of infections in future spaceflight.

## Materials and Methods

### Bacterial Strains and Culture Conditions

**Figure 1:**
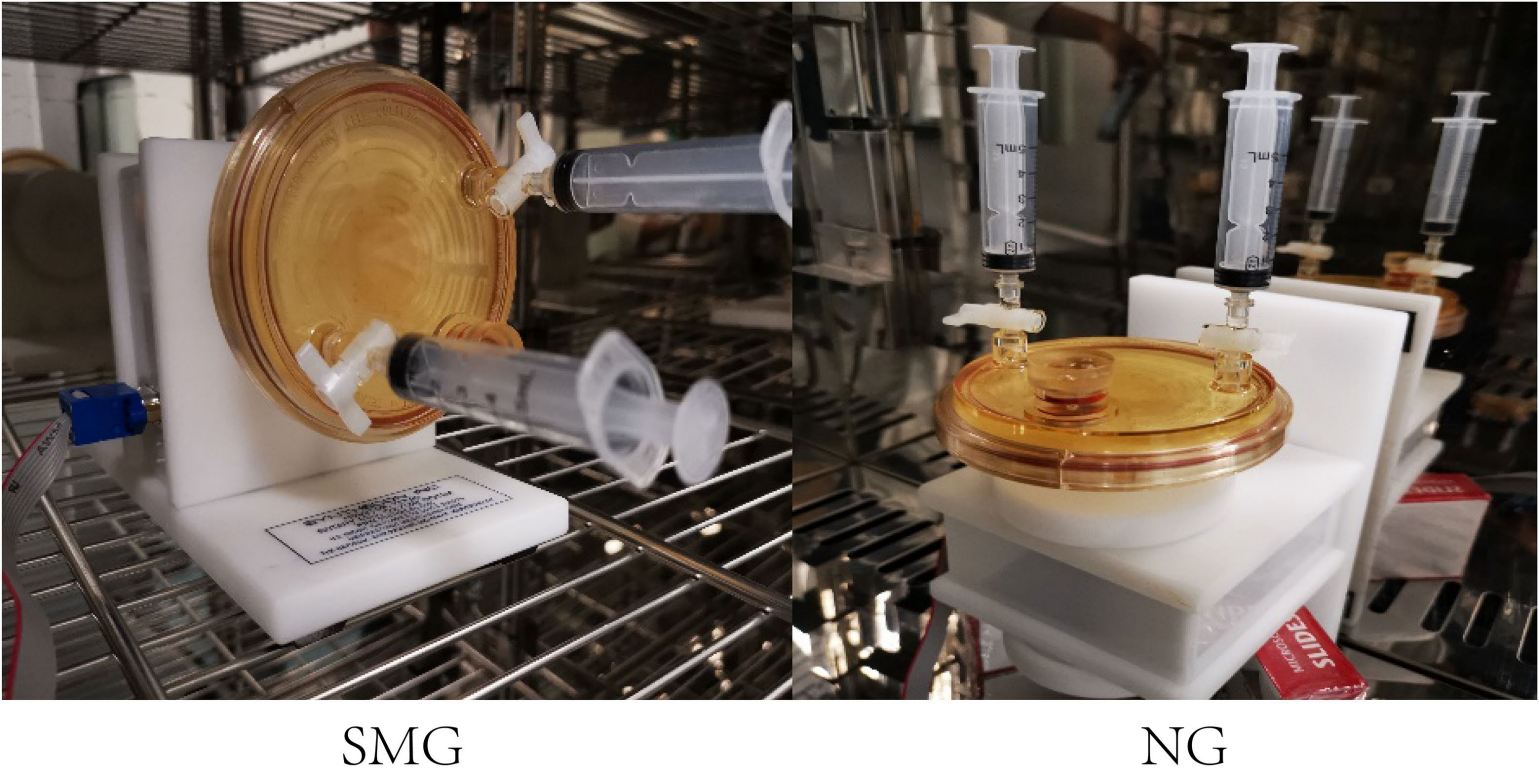
HARV bioreactors in the experimental setup. HARV system used to generate simulated microgravity (SMG) and normal gravity (NG) conditions.

The original *S. maltophilia* strain, used in this study, was purchased from the China General Microbiological Culture Collection Center. The bacterial strain was grown at 37°C in Luria-Bertani (LB) liquid medium. The original strain of *S. maltophilia* (SMO) was cultured in a shake tube containing LB liquid medium at 37°C. *Stenotrophomonas maltophilia* in simulated microgravity (SMS) and *S. maltophilia* in normal gravity (SMN) were established by growing bacteria in continuous cultivation in high-aspect rotating vessel (HARV) bioreactors (Synthecon, Inc. Houston, Tex, United States). Figure 1 shows the procedure of the SMG cultivation implemented by rotating the bioreactor on its axis perpendicular to gravity, while the normal gravity (NG) was implemented by rotating the bioreactors on its axis parallel to gravity. The overnight culture of the original strain was adjusted to a turbidity of 1.0 and then inoculated at a dilution of 1:200 in HARV bioreactors. Bubbles in the reactor were carefully removed through the valves at both ends. After incubation in HARVs for 24h at 37°C with a rotation of 25rpm, the SMS and SMN bacterial suspensions were poured out and diluted to a turbidity of 1.0, added to new HARVs in a volume ratio of 1:200 filled with LB medium, and incubated at 37°C and 25rpm for another 24h. This step of bacterial inoculation in each group was repeated 14 times for 2weeks (Wang et al., 2016b; Morrison and Nicholson, 2020).

### Scanning Electron Microscopy

Morphological changes were also observed by scanning electron microscopy (SEM), and the three groups were washed three times with sterile phosphate-buffered saline (PBS; pH 7.4) and fixed with 2.5% glutaraldehyde at 4°C overnight. The samples were washed again with deionized water three times, and each solution was settled for 7min and then dehydrated in a gradient series of ethanol (50, 70, 85, and 90%) for 15min per step. The samples were subsequently immersed in 100% ethanol (three times for 10min each) to prevent drying, and then, all the dehydrated samples were placed in a vacuum desiccator to critical point-dry. After coating the specimens with gold-palladium, the samples were observed using cold field emission scanning electron microscope (Hitachi SU8010, Japan; Haddad et al., 2021).

### Growth Curves

*Stenotrophomonas maltophilia* in simulated microgravity and SMN were taken from HARVs every 2h. Samples [10^6^ colony forming unit (CFU)/ml] were inoculated into 96-well honeycomb microtiter plates, and SMO was also prepared at the same concentration. For each group, one 96-well plate was prepared with 200μl of solution. The optical density at 600nm was measured every 2h for 24h using Bioscreen C system and BioLink software (Bioscreen C, Finland). This experiment was performed in three replicates (Chopra et al., 2006; Zhang et al., 2019).

### Carbon Source Utilization and Chemical Sensitivity Assay

Biolog GEN III plates (Biolog, Hayward, CA, United States) were used to test the ability of *S. maltophilia* to utilize 71 carbon sources and 23 chemical sensitivities in triplicate experiments. The test was performed according to the manufacturer’s instructions. Briefly, we used a sterile cotton-tipped swab to remove a bacterial colony from the surface of a BUG1B agar plate, and then inoculated it into IF-A inoculum. The concentration of the bacterial colony was adjusted to 10^8^CFU/ml by a turbidimeter (Zhang et al., 2019). A 100μl volume of inoculated IF-A was added to each of the 96 wells in the microplate. After sealing and incubating at 37°C for 24h, “negative” (−), “positive” (+), and “weakly positive” (w) were scored according to visual similarity. The endpoint data of each well were measured according to the absorbance 590nm in a microplate reader. This experiment was performed in three replicates.

### Drug Susceptibility Testing

Drug susceptibility testing (DST) was performed using the disk diffusion method based on the CLSI M100 Ed30 document (CLSI, 2020): trimethoprim-sulfamethoxazole (SXT, 25μg), levofloxacin (LEV, 5μg), and minocycline (MNO, 30μg). The entire surface of the Petri dish containing Mueller-Hinton agar (MHA) was covered with a specific inoculum (10^7^–10^8^CFU/ml; Zhao et al., 2019), and then, the plate was dried for 15min. The diameter of the zone of inhibition was measured after incubation for 18h at 37°C. All drug susceptibility tests were performed in three replicates.

### Qualitative Analysis of the Biofilm Formation Ability

After 2weeks of cultivation under SMG and NG conditions, HARVs were stained with crystal violet to evaluate the biofilm formation ability of *S. maltophilia*. Briefly, after 2weeks, the bacterial suspension was removed from the HARV bioreactors, washed gently with sterilized water twice and then stained with 0.1% crystal violet for 15min at room temperature. Subsequently, the crystal violet dye was slowly removed through a sterile syringe, the reactor was washed with sterile deionized water three times, and then, pictures were taken (Wang et al., 2017).

### Quantitative Analysis of the Biofilm Formation Ability

The SMS and SMN samples, which were cultivated for 14days in the previous step of the qualitative measurement, were collected and diluted to 10^7^CFU/ml. The SMO sample was also diluted to 10^7^CFU/ml (Zhao et al., 2019). The three samples were diluted with LB medium at a volume ratio of 1:100, and added to a 24-well plate. Each well contained 1ml LB medium. Then, the 24-well plate was sealed and fixed with rubber bands and placed in an oscillation incubator at 37°C and 200rpm for 24h. Next, planktonic bacteria were removed. After washing each well with deionized water twice, the plate was placed in a 50°C constant temperature incubator to dry for 15min to fix the biofilm. About 2ml of 0.1% crystal violet dye was added to each well at room temperature for 15min to stain the adherent biofilm. Then, crystal violet was removed, and the biofilm was slowly washed twice with sterile deionized water, followed by the addition of 2ml DMSO to fully digest the biofilm. Finally, the suspension was transferred to a 96-well plate to measure OD_570_. The results are presented as the mean±SD of three biological replicates. In addition, the SMS and SMN samples obtained from the cultivation after 14days in HARV bioreactors were diluted to 10^7^CFU/ml and immediately subjected to CFU assay to identify the differences in viability (Shui et al., 2021).

### Analysis of the Biofilm Formation Ability *via* Confocal Laser-Scanning Microscopy

The biofilm formation ability of the three groups was assessed using Filmtracer™ (LIVE/DEAD Biofilm Viability Kit, L10316, Molecular Probes, Invitrogen, CA, United States) in conjunction with confocal laser-scanning microscopy (CLSM; Leica TCS SP8, Berlin, Germany). LIVE/DEAD Biofilm Viability is a culture-independent method that depends on the cell membrane integrity as a viability measure; it utilizes a mixture of the SYTO9 green fluorescent nucleic acid stain and a red fluorescent nucleic acid stain and propidium iodide. Live and dead bacteria can be distinguished without waiting for growth plate results. The working solution of the fluorescent stains was prepared by adding 3μl of the SYTO9 stain and 3μl of the propidium iodide stain to 1ml filter-sterilized water. The excitation/emission maxima for these dyes are approximately 480/500nm for SYTO9 staining and 490/635nm for propidium iodide. Three independent rounds of biofilm experiments were performed. Briefly, in each round of experiments, SMS, SMN, and SMO were inoculated in 35-mm confocal dishes (Solarbio, Beijing, China), cultivated at 37°C for 24h, and then rinsed two times with sterile water to eliminate nonadherent bacteria. Then, the generated biofilms were carefully stained with 200μl working solution stain for 30min in the dark at room temperature. It was important to immediately add the stain before the biofilm dried. After removing the stain and washing two times with sterile water, images were acquired from random positions, and the number of images from each layer varied according to the thickness of the biofilm. The maximum biofilm thickness of the uniform position in the current field of view was set as the biofilm thickness (Jiang et al., 2021; Shui et al., 2021).

### Motility Assay

To assess the motility of *S. maltophilia*, the SMS and SMN samples were taken from HARV bioreactors immediately after 14days of SMG and NG cultivation. The difference in swimming motility was assessed using agar plates with slight modifications (Murray and Kazmierczak, 2006). Briefly, to monitor swimming, *S. maltophilia* diluted to 10^7^CFU/ml was point inoculated with a sterile inoculation needle onto plates containing 0.1% (w/v) Bacto agar, 10g/L Tryptone (w/v), and 5g/L Yeast extract (w/v). The inoculated samples were incubated for 48h at 37°C, the plates were imaged, and the diameter of the zone of growth around the spot inoculation was measured. All the motility tests were performed in three replicates.

### Genome Sequencing

#### Genome Sequencing and Assembly

DNA from the original strain of *S. maltophilia* was acquired using the conventional phenol–chloroform extraction method (Javadi et al., 2014). After the genomic DNA was segmented by Covaris, a genomic sequencing library was constructed. The whole genome was sequenced using the Illumina Hiseq and PacBio platforms according to standard quality controls, and raw reads were obtained. The raw reads were then filtered using the following steps: the adapter sequence was removed, bases other than A, G, C, and T at the 5'-end were removed, bases containing “N” reaches above 10% were removed; the ends of reads with lower sequencing quality (<Q 20) were trimmed; and the adapters and quality-trimmed small fragments less than 25bp in length were removed. Self-correction was carried out using Falcon software (Version 0.3.0), and corrected reads were acquired. Quality assessment of the genome sequence obtained from the preliminary assembly determined whether the genome was contaminated, assessed the quality of sequencing, and assessed the condition of the genome. The bacterial genome scan map was assembled using SOAPdenove2 software, and multiple k-mer parameters were spliced after the sequences were optimized. Then, the optimal contig assembly results were obtained. The reads were compared to the contigs to form scaffolds according to the relationship between paired-ends and overlaps. The map of the complete bacterial genome was assembled using the canu and SPAdes. Overlaps of a certain length at both ends of the final assembly sequence were cut off at one end. Finally, the complete chromosome sequence was obtained (Lu et al., 2019; Jiang et al., 2020; Table 1).

**Table 1:** The assembly data of SMO.

Sequence type	Sequence topology	Sequence number	Plasmid No.	Total length (bp)	GC content (%)
Chromosome	Circular	1	0	4,779,890	66.37

#### Genome Component Prediction

The genome component prediction included the prediction of coding genes, transfer RNA (tRNA), ribosome RNA (rRNA), and tandem repeat sequences. The detailed steps were performed as follows: (a) the related coding genes were predicted by Glimmer, GeneMarks and Prodigal software; (b) tRNA genes and rRNA genes were recognized by tRNAscan-SE software (Version V2.0) and Barrnap software, respectively. The nucleotide sequence information, anticodon information, and secondary structure information of the tRNA in each sample genome were obtained; and (c) Tandem repeat annotations were retrieved by the Tandem Repeats Finder. The type, location, and sequence information of all the rRNAs in the genome of each sample were obtained (Cao et al., 2020).

#### Gene Function Analysis

Six databases containing NR (Non-Redundant Protein Database), SwissProt, Pfam, COG (Clusters of Orthologous Groups), GO (Gene Ontology) and KEGG (Kyoto Encyclopedia of Genes and Genomes) were used for general gene function annotation. A whole-genome BLAST alignment was constructed using the six databases.

#### Comparative Genomic Analysis

The genomes of SMS and SMN were sequenced using high-throughput sequencing Illumina technology (Chi et al., 2021; Hao et al., 2021). In brief, DNA was extracted from samples of SMS and SMN and randomly fragmented, and DNA fragments of a certain length were recovered by electrophoresis. Then, DNA fragments assembled with adapters were amplified to prepare DNA clusters, and the clusters were sequenced (Gao et al., 2020; Zhang et al., 2021).

The sequences obtained using fastq software (Version 0.20.0) were filtered by removing reads that contained adapters and poly-N as well as those with a lower sequencing quality (<Q 20). The original SMO group was used as a reference. The SMS and SMN reads were mapped to that of the reference sequence using the mem algorithm of BWA software, and the coverage was determined using Picard-tools. Then, according to the comparison bam results, the sequencing depth and relative coverage were acquired. GATK was used to realign the reads near InDels to obtain the realigned bam, which could eliminate false positive single-nucleotide polymorphisms (SNPs). Then, snippy software (Version 4.6.0) was used to detect SNPs and small InDels. Finally, snpEff was used to annotate variant sites to determine the impact of mutations on the genome (Jiang et al., 2020).

### Transcriptome Sequencing and Comparative Transcriptomic Analysis

#### Sequencing and Filtering

RNA was isolated immediately from bacterial cells incubated in semisolid LB medium. About 2μg RNA was obtained from the two groups using a TruSeqTM Stranded Total RNA Library Prep Kit (Illumina, United States) following the manufacturer’s instructions. Around 2 μg of RNA from each sample was used for RNA sample preparation after the concentration and purity of the RNA was detected by a Nanodrop 2020. After removing rRNAs, the purified mRNAs were fragmented into small pieces (~200bp) using fragmentation buffer. The RNA fragments were used to produce first strain cDNA by reverse transcriptase PCR with random primers. Then, second strain cDNA was created using dUTP instead of dTTP. The sticky end of the double strand cDNA was linked to the adaptor and digested with the UNG enzyme. Then, the cDNA fragments were enriched *via* PCR amplification and quantified by TBS380 (Picogreen), and the clusters were sequenced using the Illumunia Hiseq platform (An et al., 2020).

### Gene Expression Statistics and Differential Gene Expression Analysis

The quantitative expression software RSEM was used to quantitatively analyze the expression levels of genes and transcripts. The transcripts per million (TPM) and reads per kilobase million (FPKM) of each gene were calculated in accordance with gene length, and the regulation mechanism of genes were revealed by combining the sequence function information. According to the expression of genes in different samples, Venn, correlation and principal component analysis (PCA) analyses were performed. Venn analysis was used to display genes that were shared and uniquely expressed between samples. Correlation analysis of the biological replicate samples, on the one hand, checked whether the variation between biological replicates met the expectations of the experimental design, and on the other hand, provided a basic reference for the analysis of differentially expressed genes (DEGs). PCA could reduce the complexity of the data and determine the relationship and variation between samples. Finally, the read counts were mapped to the genes. Differential gene expression analysis was analyzed using DESeq2 software, and genes yielding a value of *p*<0.05 and fold change (FC) >2 in two different samples were identified as DEGs (Zhang et al., 2020).

### Functional Annotation and Enrichment Analysis

The identified DEGs were annotated on the basis of COG, GO, and KEGG functional annotation. The gene lists were enriched according to GO and KEGG enrichment analyses. GO enrichment analysis of the DEGs was carried out using Goatools software. GO terms with a value of *p*<0.05 were identified as significantly enriched by DEGs. R scrip was used to estimate the meaningful statistical enrichment of the DEGs in KEGG pathways (Wang et al., 2020).

### Proteomics Analysis

#### Polypeptide Preparation

Frozen bacterial samples stored in −80°C were transferred to shaking tubes, and lysis buffer was added to the samples and mixed well (Kan et al., 2020). A high-throughput tissue grinder was used and shaken three times for 40s each time. The suspension was placed on ice for 30min and sonicated every 5min for 10s. After incubating at 100°C for 10min, the samples were refrigerated on ice, and centrifuged at 12,000*g* for 20min at 4°C. Precooled acetone was added to the supernatant at a ratio of 1:4 and precipitated overnight at −20°C. The next day, the mixture was centrifuged at 12,000*g* for 20min, and then, the supernatant was discarded. Next, 90% precooled acetone was added to the pellet and mixed well. This step was repeated twice. Then, the protein lysis solution was added to the precipitate, and after centrifugation at 12,000*g* for 20min at 4°C, the supernatant was obtained. The proteins were quantified by BCA and subsequently confirmed using SD-PAGE. After reductive alkylation and enzymatic hydrolysis, the processed peptides were labeled with Tandem Mass Tag™ (TMT™), reconstituted with UPLC loading buffer and subjected to high-pH liquid fractionation with a reversed-phase C18 column (Thermo, United States). Then, the purified peptides were subjected to an EASY-nLC 1,200 instrument (Thermo, United States) and Q_Exactive HF-X (Thermo, United States) to obtain raw data. The raw data were converted into MGF format by the Thermo Scientific tool Proteome Discoverer software (Version 2.4), and the exported MGF files were searched against the established database using the Sequest and Mascot modules. Eventually, analyses of the data and bioinformatics were performed according to the previous search results (Dai et al., 2020; Chen et al., 2021; Ye et al., 2021).

### Functional Annotation and Differential Protein Analysis

According to the mass spectrometry results, all proteins and sequences were compared with information in databases, including GO, KEGG, COG, and subcellular location related databases, to obtain the annotation information of the proteins in each database and statistics on the annotations. Proteins with a fold change >1.2 or <0.83 and value of *p*<0.05 in two different groups were defined as differentially expressed proteins (DEPs). Additional protein lists were assembled based on the DEPs, including GO, KEGG, and COG functional annotation and GO and KEGG pathway enrichment analyses.

## Statistical Analysis

Differences in the growth rates among SMS, SMN, and SMO were evaluated by two-way ANOVA, followed by Tukey’s multiple comparisons test. The biofilm formation ability and motility were evaluated by unpaired *t*-tests. GraphPad Prism (version 8.4.3) was used for statistical analysis. Differences with values of *p*<0.05 were considered significant.

## Results

### Morphology

**Figure 2:**
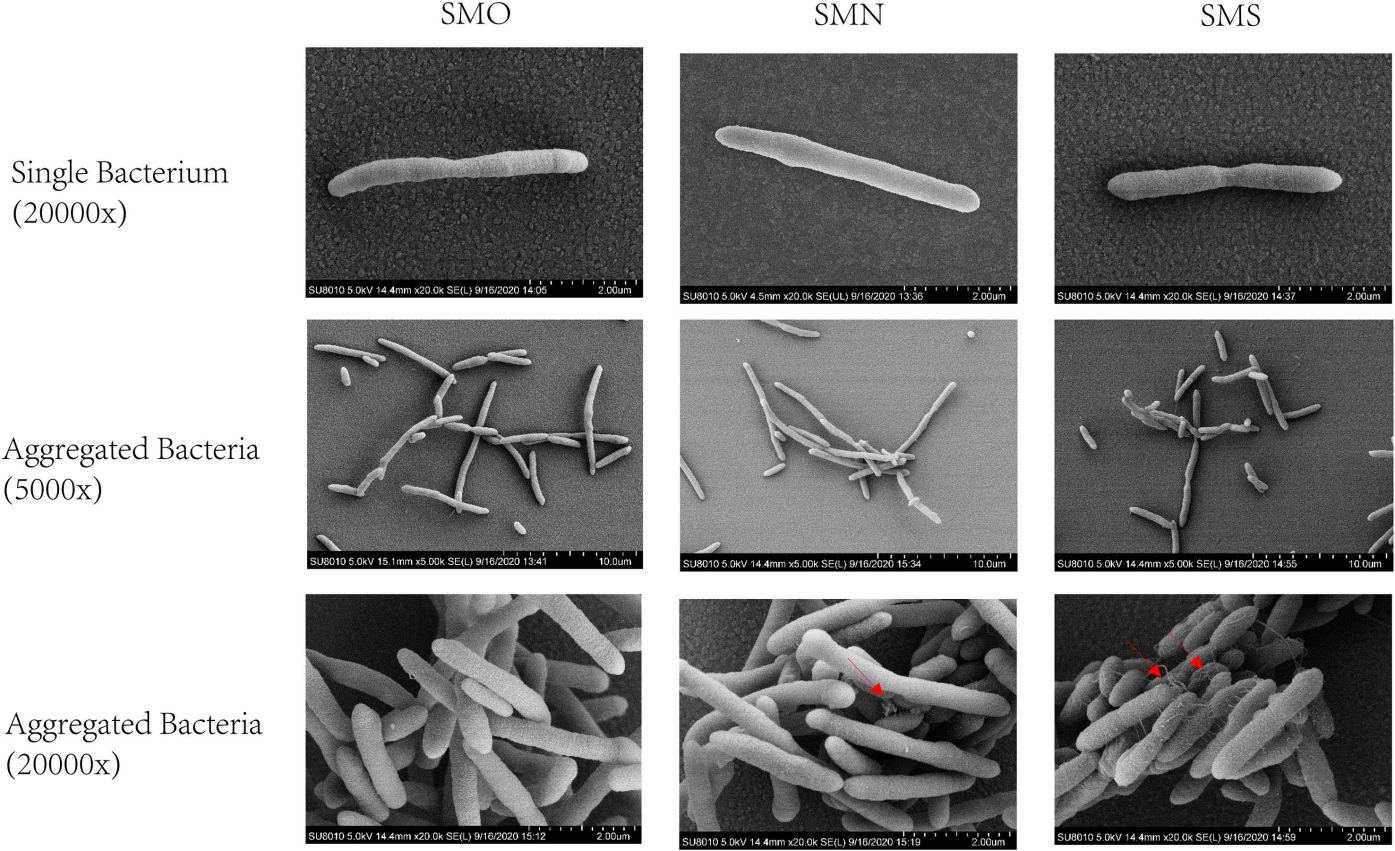
Scanning electron micrographs (SEM) images of *Stenotrophomonas maltophilia*. The red arrow indicates the intercellular mucus-like substances. The **first row** showed the morphology of single bacterium under 20,000x SEM. The **middle row** showed the morphology of aggregated bacteria under 5,000x SEM. The **last row** showed the morphology of aggregated bacteria under 20,000x SEM.

Scanning electron microscopy was performed to observe the single cell morphology of SMO, SMN, and SMS. From observing the single bacteria, most of the three groups were found to be long rod shape. Under 5,000x SEM, the three groups showed entanglement and aggregation, and some shorter bacilli appeared sporadically. Under 20,000x SEM, it was found that SMO was full and smooth, but there were unequal mucus-like substances in SMN and SMS, and SMS had more mucus-like substances than SMN (Figure 2).

### Growth Rate Assay

**Figure 3:**
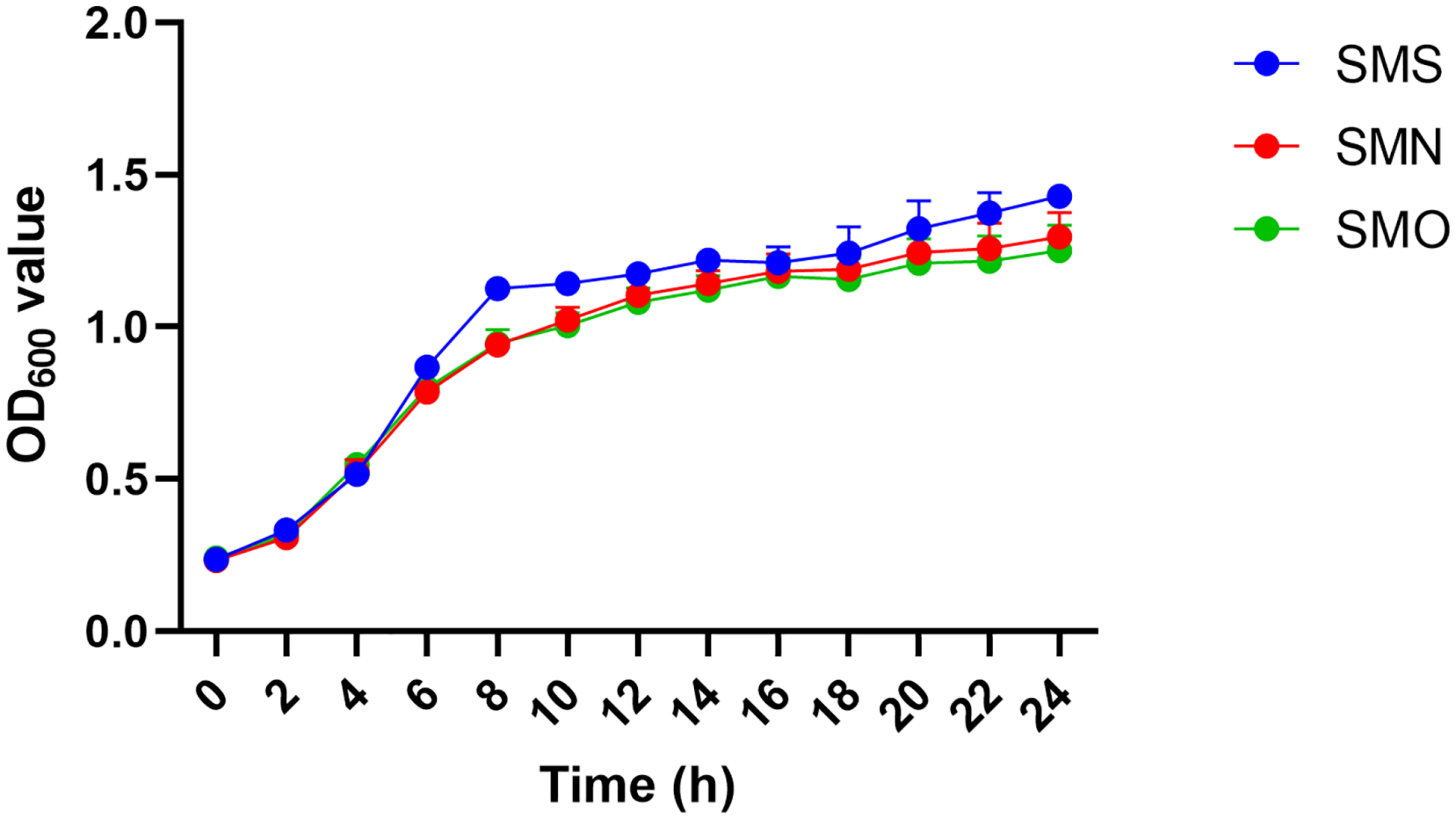
Growth curves of three *S. maltophilia* samples. Growth curves of *Stenotrophomonas maltophilia* in simulated microgravity (SMS; blue), *S. maltophilia* in normal gravity (SMN; red), and original strain of *S. maltophilia* (SMO; green) were determined by measuring the OD_600_ value.

Growth rate curves for the three groups are shown in Figure 3. Compared with the SMN group, the SMS group exhibited a mild increased growth rate, especially after 6h (*p*<0.0001), 8h (*p*=0.0004), and 10h (*p*=0.011), while the SMO showed a similar growth curve to that of the SMN group.

#### Carbon Source Utilization Assays and Chemical Sensitivity and Drug Susceptibility Testing

Compared with SMO and SMN, SMS showed decreased carbon source utilization of D-arabitol (D3), myo-inositol (D4), D-aspartic acid (D8), L-pyroglutamic acid (E8), quinic acid (F8), and D-lactic acid methyl ester (G3), indicating that the metabolism pathways of SMS were inhibited in the SMG environment. In addition, the chemical sensitivity assay showed decreased tolerance to potassium tellurite (G12; Table 2). SMS, SMN, and SMO were all sensitive to SXT, LEV, and MNO. The drug susceptibility test results showed no statistically significant difference in the inhibitive zone diameter of the three antibiotics.

**Table 2:** Biochemical characteristics of three *Stenotrophomonas maltophilia* samples.

Substrate	SMO	SMN	SMS
D-arabitol (D3)	+	+	w
myo-inositol (D4)	+	+	w
D-aspartic acid (D8)	+	+	w
L-pyroglutamic acid (E8)	+	+	w
quinic acid (F8)	+	+	w
D-lactic acid methyl ester (G3)	+	+	−
potassium tellurite (G12)	+	+	−

*“+” represents a positive reaction; “−” represents a negative reaction; “w” represents a weakly positive reaction*.

### Biofilm Assay

**Figure 4:**
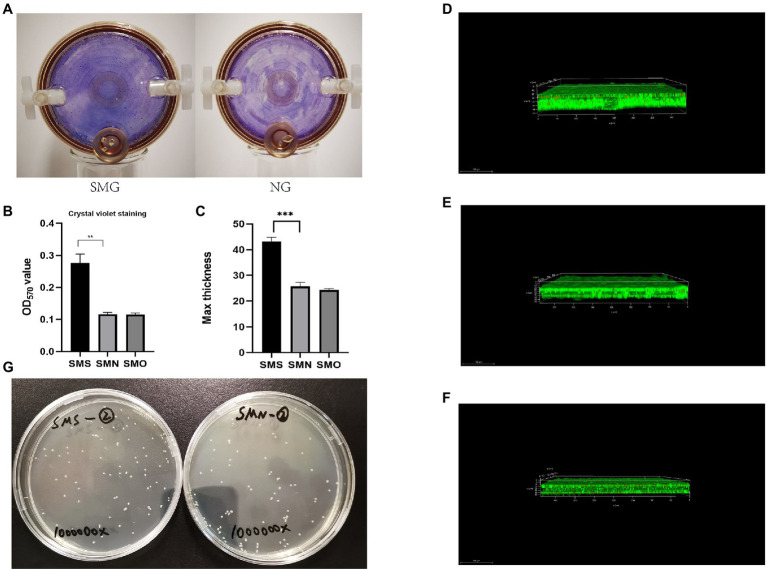
Biofilm formation assay. **(A)** Quantitative analysis of biofilm formation ability *via* crystal violet. The three groups were cultured in 24-well plate at 37°C and 200rpm for 24h. Biofilm formation ability was measured by determining the OD_570_ of crystal violet. **(B)** Quantitative analysis of biofilm formation ability *via* crystal violet. The three groups were cultured in 24-well plate at 37°C and 200rpm for 24h. Biofilm formation ability was measured by determining the OD570 of crystal violet. **(C)** Biofilms of SMS, SMN, and SMO cultured in 35-mm confocal dished. Cells were stained with a Filmtracer™ (LIVE/DEAD Biofilm Viability Kit), and biofilm formation ability was detected using confocal laser-scanning microscopy (CLSM). Green fluorescence indicates live cells and red fluorescence indicates dead cells. The maximum thickness of biofilm of SMS, SMN, and SMO were (43.17±2.73; 25.75±1.59), and (25.36±1.56) μm, respectively. ^**^p<0.01 and ^***^p<0.001. **(D)** Analysis of biofilm formation ability of SMS samples using CLSM. **(E)** Analysis of biofilm formation ability of SMN samples using CLSM. **(F)** Analysis of biofilm formation ability of SMO samples using CLSM. **(G)** Colony forming unit (CFU) assay of SMS and SMN samples.

The phenotypes of biofilm formation under both the SMG and NG conditions were analyzed after 2weeks of cultivation by observing the films that generated and adhered to the gas-permeable membranes at the bottom of the bioreactors. The biofilm formed by *S. maltophilia* grown in the SMG environment were thicker than those grown in the NG environment (Figure 4A). The biofilm formation ability of the three groups was quantified by the OD_570_ values of crystal violet staining. The biofilm formation ability of SMS was more than twice that of SMN (*p*=0.0006; Figure 4B). There was no significant difference between SMN and SMO. Besides, colony forming unit analysis of viable counts of bacteria was performed to identify the differences in viability. The result showed that there was no statistically significant between SMS (97.6±4.4) and SMN (96.3±2.0) samples after 10^6^ times dilution (*p*=0.5286; Figure 4G). Furthermore, the biofilm formation ability results were confirmed *via* CLSM analysis. The image stacks obtained from each sample illustrated that the maximum biofilm thickness of SMS (Figure 4C) was significantly greater than that of both SMN (*p*=0.0002; Figure 4D) and SMO (*p*<0.0001; Figure 4E). There was no significant difference in biofilm thickness between SMN and SMO (Figure 4F).

### Mobility

**Figure 5:**
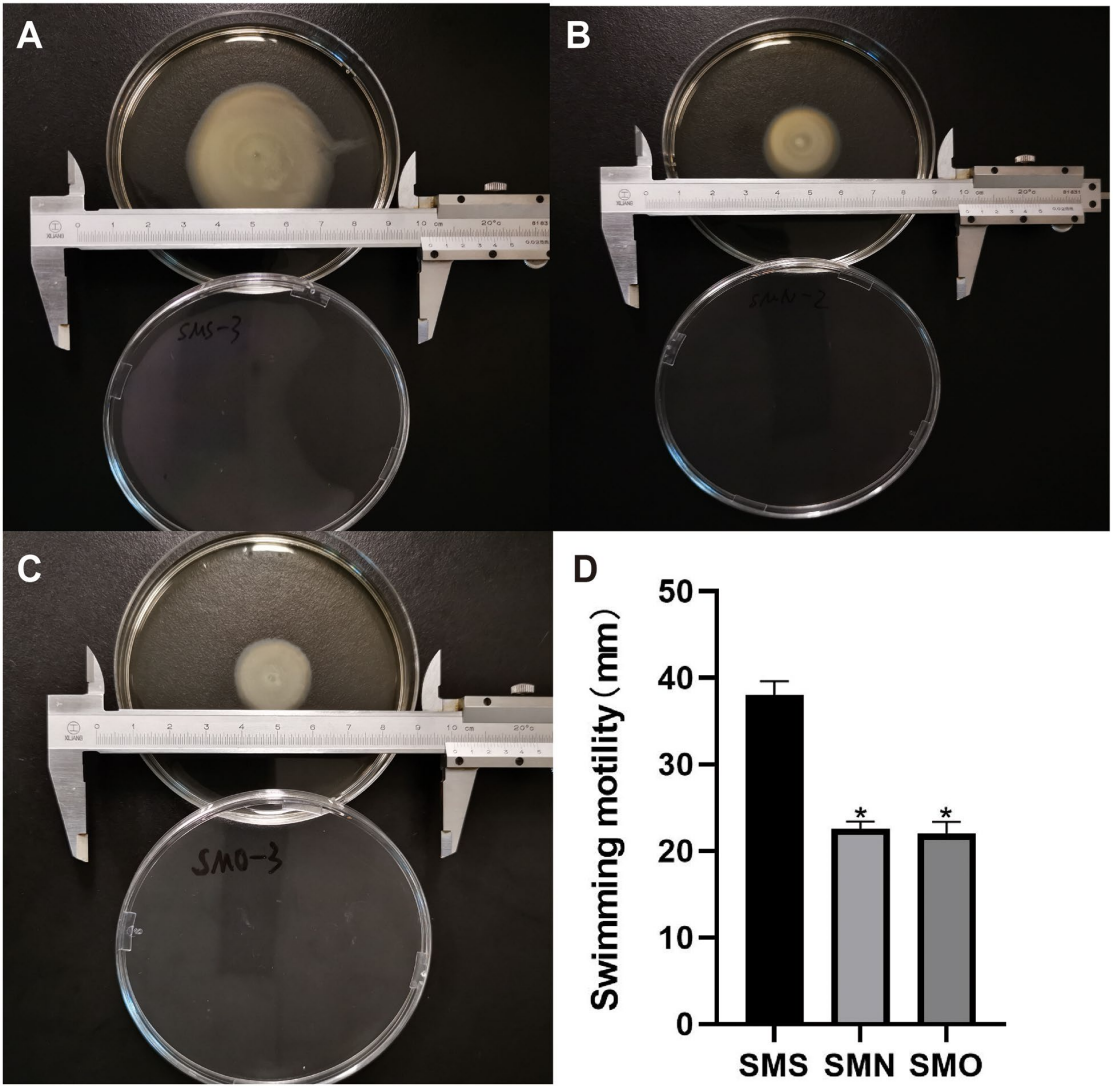
Effect of SMG on mobility of *S. maltophilia*. For the swimming motility, three groups were inoculated on a surface of 0.1% (w/v) Bacto agar plates. Swimming haloes were gauged after 48h of incubation at 37°C. Zone diameters (means±SD; *n*=3) are listed for each group. **(A)** SMS (37.96±1.34mm), **(B)** SMN (22.56±0.68mm), and **(C)** SMO (21.96±1.13mm). **(D)** Asterisks (^*^) indicate statistically significant change (*p*<0.05) compared to that of SMS group. ^*^p<0.05.

All three groups were able to move on the interface between the bottom of the plate and the medium in our study. On the swimming plates, SMS formed round and translucent colonies with dense cells at the inoculation site. The average colony diameter of SMS was 37.96±1.34mm (*n*=3). However, the colony diameters of SMN and SMO were 22.56±0.68mm (*n*=3), and 21.96±1.13mm (*n*=3) respectively, both of which were smaller than that of SMS, and the difference was statistically significant (*p*=0.0066, *p*=0.0057; Figure 5).

### Genome Sequencing, Assembly, and Annotation

**Figure 6:**
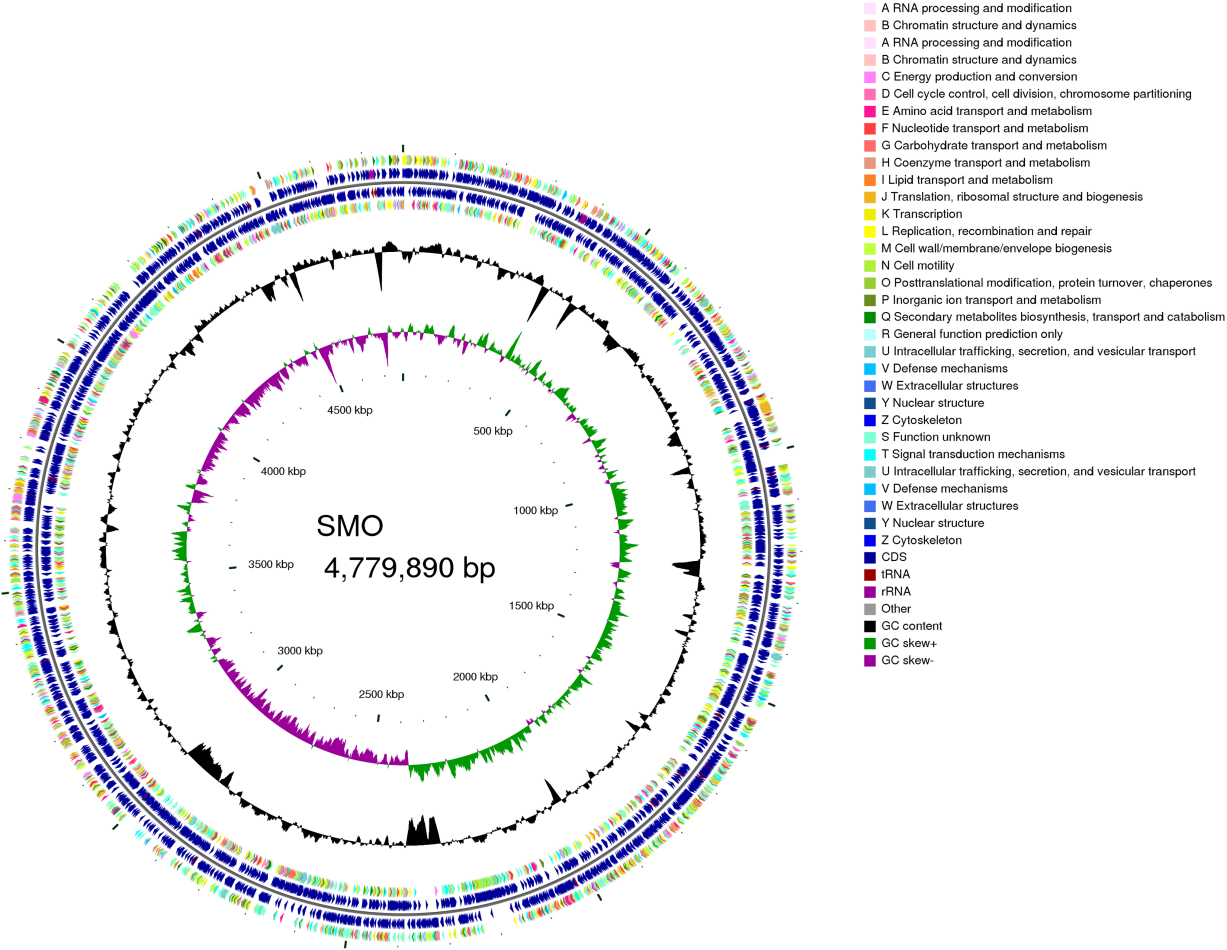
Genome map of SMO. From outer to inner circle: CDS on the positive-strand, different colors indicate different Clusters of Orthologous Groups (COG) function classifications (ring 1), CDS, transfer RNA (tRNA), ribosome RNA (rRNA) on the positive-strand (ring 2), CDS, tRNA, and rRNA on the negative-strand (ring 3), CDS on the negative-strand, different colors indicate different COG function classifications (ring 4), GC content (ring 5), GC-Skew (ring 6).

The accession number of SMO is SRR14180817. The draft genome of SMO was estimated to be 4.78 Mbp with a GC content of 66.37%. In addition, SMO was predicted to have 4,437 coding sequences, 74 tRNAs, 13 sRNA, and seven gene islands. The average length of the coding genes was 941.55bp, encompassing 87.40% of the genome. More information about the genome is shown in the circular map (Figure 6).

### Comparative Genomic Analysis

The accession number for the resequencing data of the SMS is SRR14181238, and that for SMN is SRR14181566. SMO was used as a reference strain, and SMN and SMS were mapped to the genome of SMO to estimate genomic changes. One indel was found between SMN and SMO, including 10 related genes in coding regions and one related gene in an intergenic region. One indel was found between SMS and SMO, including 10 related genes in coding regions and one related gene in an intergenic region. In addition, one SNP was found between SMS and SMO, including eight related genes in coding regions and one related gene in an intergenic region. The reference base “G” was mutated to “C,” which attributed to transversion mutation, the related upstream and downstream genes were mostly about ribosomal protein and ATP-binding protein. The details of the indels and SNP located in the CDS are presented in Table 3.

**Table 3:** Summary of all indels and single-nucleotide polymorphisms (SNPs) located in the CDS of SMS and SMN.

Strain	Type	Gene ID	Annotation
SMS and SMN	Deletion	Gene3316	Peptidase M20
	Deletion	Gene3321	Hypothetical protein
	Deletion	Gene3322	Two-component system response regulator
	Deletion	Gene3313	Drug resistance transporter
	Deletion	Gene3314	NADPH-dependent ferric siderophore reductase
	Deletion	Gene3315	Hypothetical protein
	Deletion	Gene3317	Hypothetical protein
	Deletion	Gene3318	Phosphoethanolamine transferase
	Deletion	Gene3319	Hypothetical protein
	Deletion	Gene3320	Hypothetical protein
SMS	SNP	Gene1305	ABC transporter ATP-binding protein
	SNP	Gene1310	Lipid II flippase MurJ
	SNP	Gene1311	Bifunctional riboflavin kinase/FAD synthetase
	SNP	Gene1312	tRNA ligase
	SNP	Gene1306	50S Ribosomal protein L21
	SNP	Gene1307	50S Ribosomal protein L27
	SNP	Gene1308	Redox-regulated ATPase YchF
	SNP	Gene1309	30S Ribosomal protein S20

### RNA-Seq Alignment and Comparative Transcriptomic Analysis

The accession number of the transcriptomic data for SMS is SRR14193993, and that for SMN is SRR14183401. The sequencing reads of SMS and SMN were mapped to the reference genome of SMO. The percentages of the total reads for SMS and SMN mapped to the reference strain were approximately 97.85 and 98.47%, respectively. The uniquely mapped reads of SMS and SMN were 92.90 and 94.68%, respectively.

**Figure 7:**
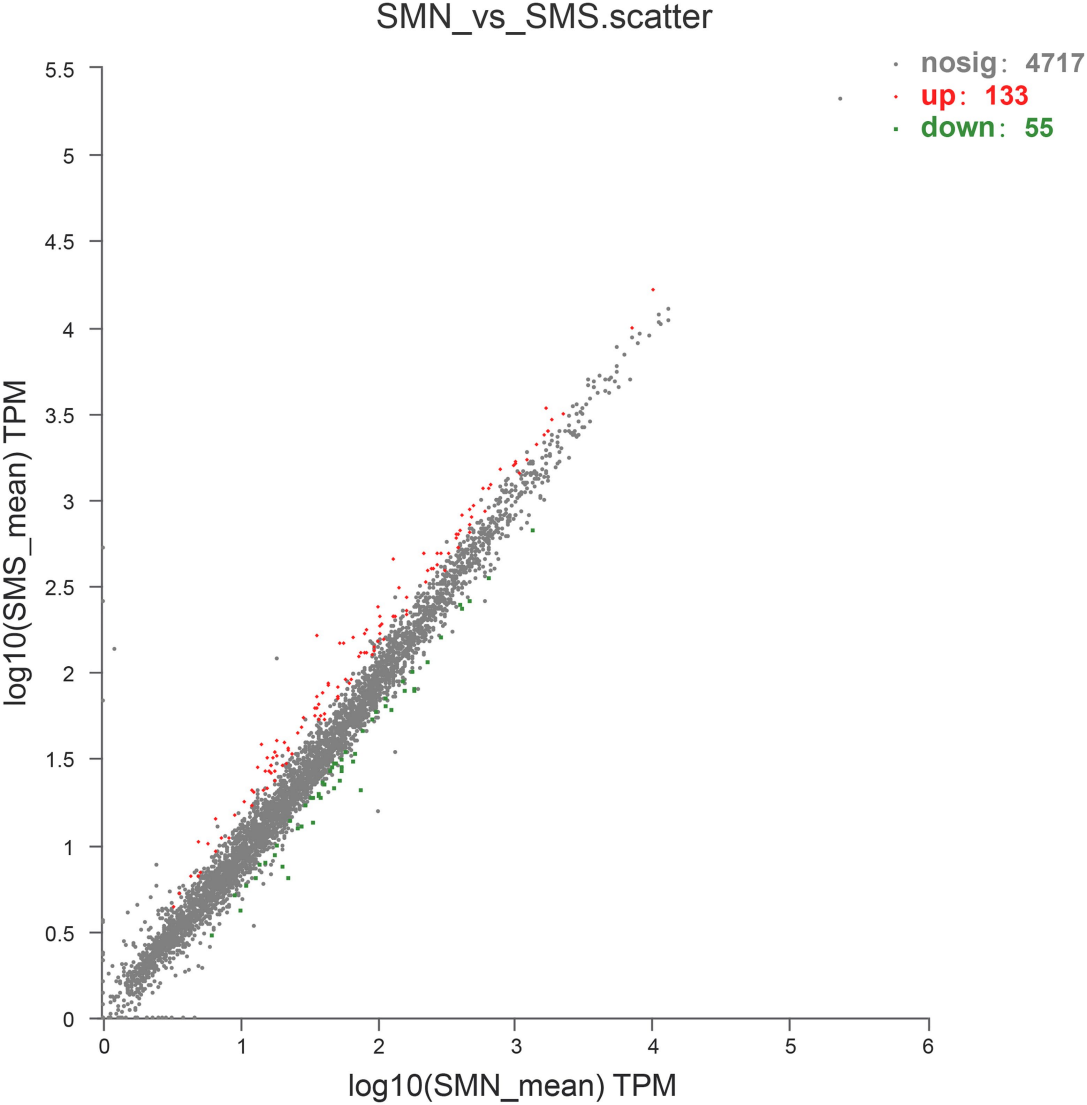
Upregulated and downregulated genes expressed in SMS compared with that in SMN. The values of the abscissa and ordinate have been algorithmized, and each point represents a specific gene. The abscissa corresponding to a specific point is the expression level of the gene in SMN, and the ordinate is the expression level of the gene in SMS.

**Figure 8:**
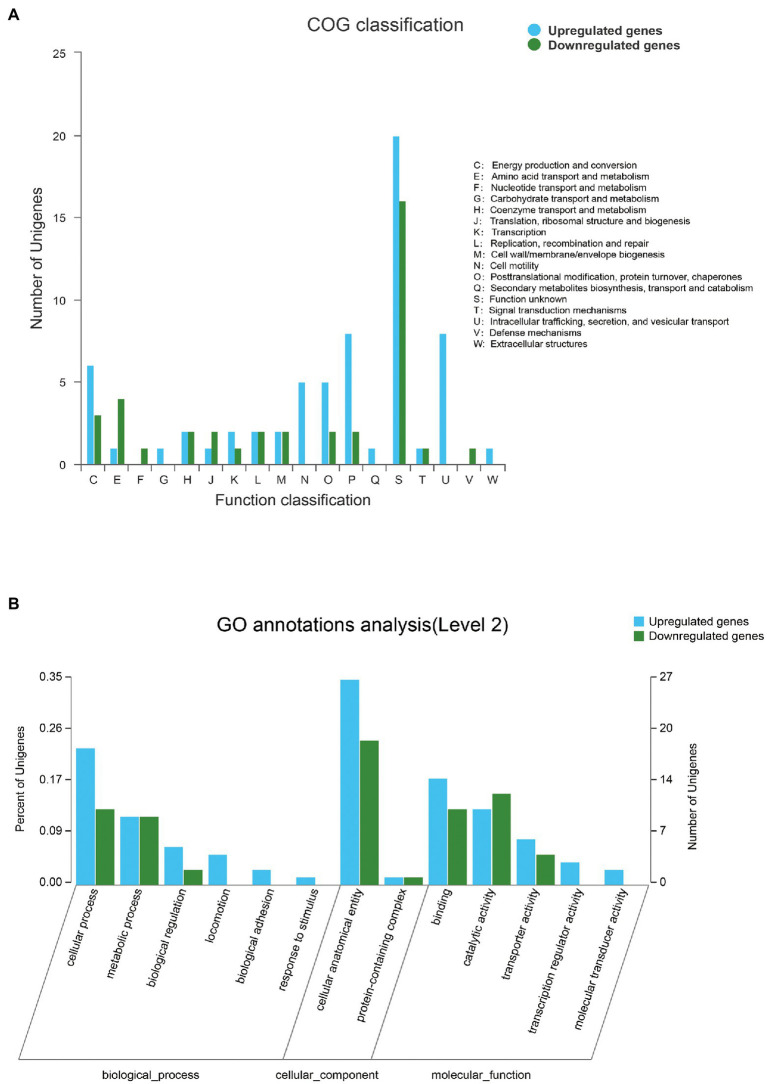
**(A)** Distribution of differentially expressed genes (DEGs) between SMS and SMN in the cluster of COG. The X-axis represents the functional type of COG, and the *Y*-axis represents the number of genes. **(B)** Distribution of DEGs in Gene Ontology (GO) functional categories. The X-axis represents the secondary classification terms of GO, the *Y*-axis on the left represents the percentage of genes included in the secondary classification, the right represents the number of genes compared with the secondary classification, and the three colors represent the three major classifications.

Overall, 188 DEGs were identified between SMS and SMN according to the TPM reads values. Compared with SMN, SMS contained 133 upregulated and 55 downregulated genes (Figure 7). In addition, 115 DEGs were mapped to COG categories, most of which were associated with cell motility, intracellular trafficking, secretion, vesicular transport, energy production and conversion, and inorganic ion transport and metabolism (Figure 8A). The pilP, pilM, flgE, flgG, and ronN genes, which are associated with cell motility, were upregulated. The pilA, pilO, pilQ, and ata genes, which are associated with fimbrial protein and outer membrane protein formation, were also upregulated.

Differentially expressed genes were also mapped according to the results of GO function analysis (Figure 8B). The DEGs were enriched in cellular components (CCs), molecular function (MF), and biological processes (BPs). For CCs, the DEGs between SMS and SMN were classified into two types. For MF, the DEGs were classified into five types, and six types were included in BPs. A total of 77 genes were annotated in the three categories, most of which were associated with cellular anatomical entities, binding, catalytic activity, and cellular processes. It is worth noting that the same DEGs probably existed in different categories.

**Figure 9:**
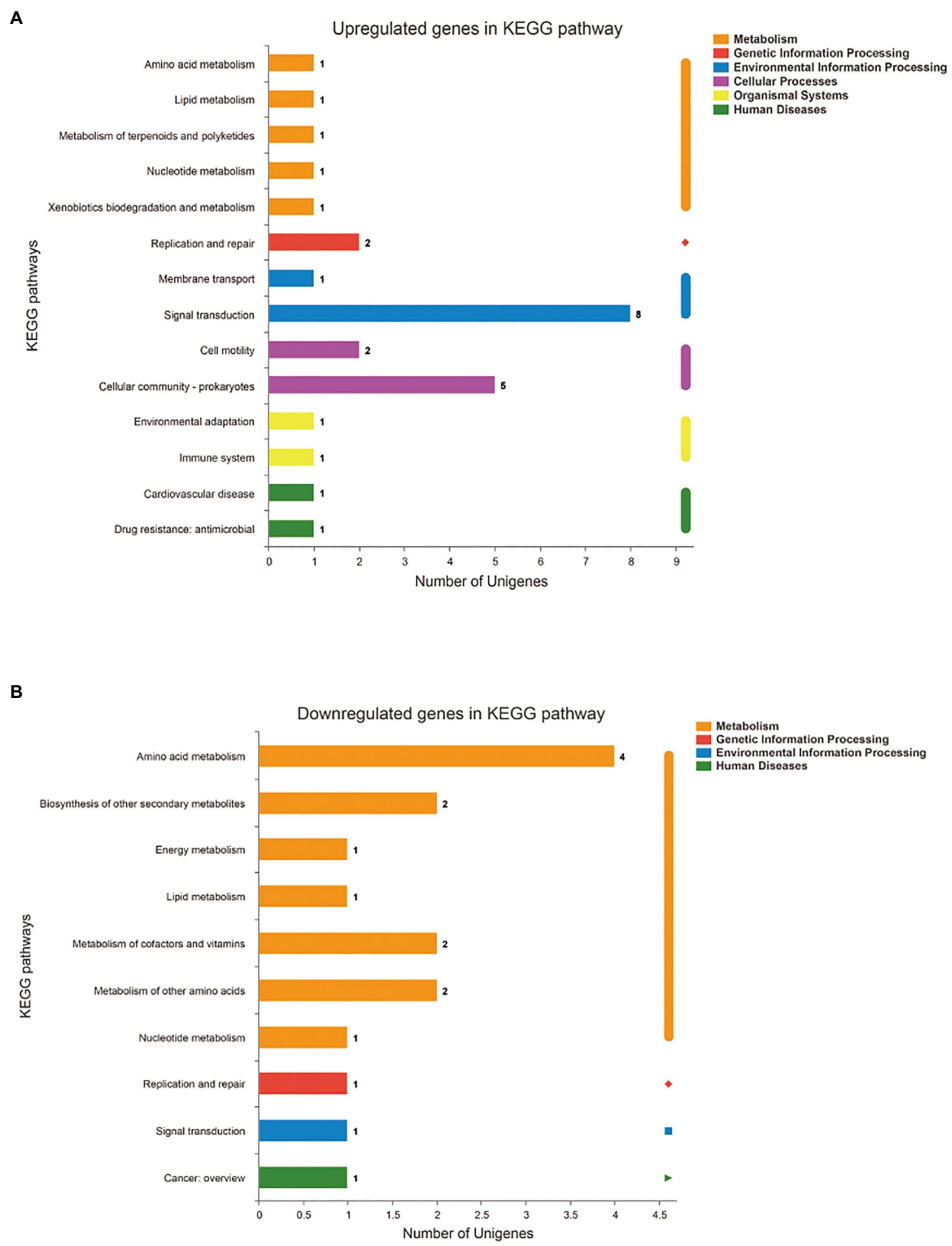
Functional annotation and enrichment of genes of SMS. **(A)** Kyoto Encyclopedia of Genes and Genomes (KEGG) pathway of upregulated genes in SMS. **(B)** KEGG pathway of downregulated genes in SMS. The X-axis of **(A)** and **(B)** is the number of genes annotated to the KEGG pathway, the Y-axis of **(A)** and **(B)** is the name KEGG metabolic pathway.

In addition, the differentially expressed genes were annotated and enriched in the KEGG pathway database. In total, the 43 DEGs included in the first six categories and 19secondary biochemical categories or branches of KEGG metabolic pathways, were statistically significant between the SMS and SMN groups, most of which are associated with signal transduction, cellular community, cell motility, and amino acid metabolism (Figures 9A,B).

### Comparative Proteomic Analysis

**Figure 10:**
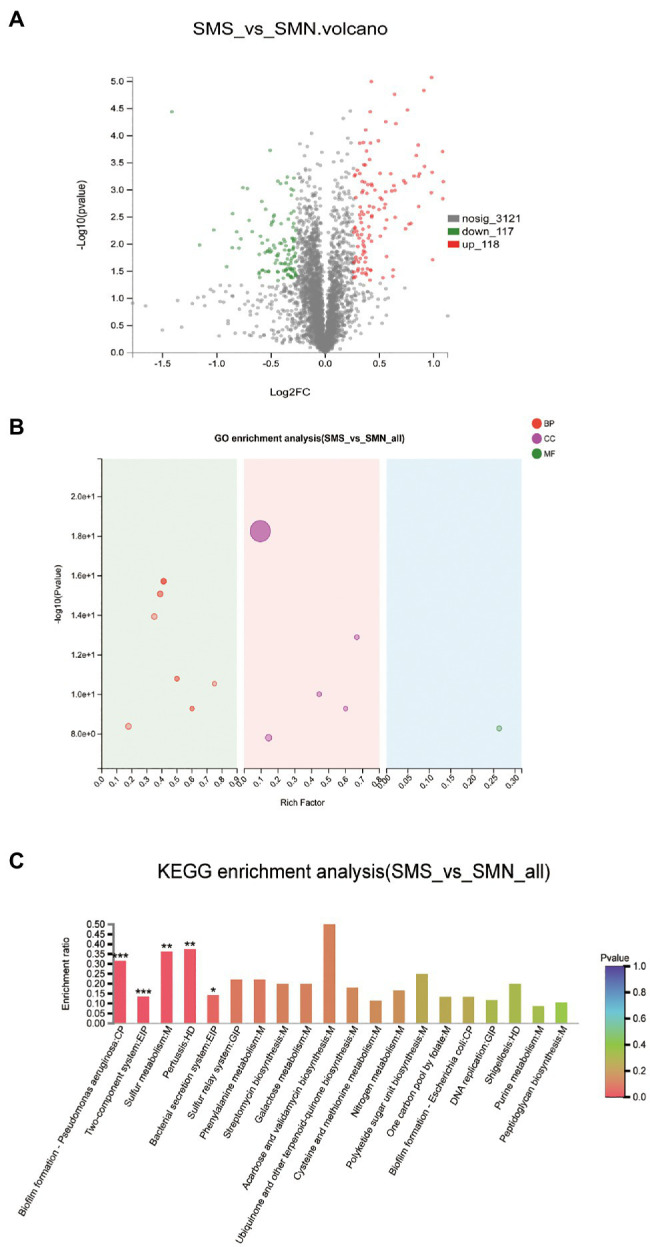
Comparative proteomic analysis. **(A)**. Differentially expressed proteins (DEPs) between SMS and SMN. The X-axis is the fold change value of the difference in the protein expression between the two groups. The Y-axis is the statistical test value of the DEPs. The value of abscissa and ordinate have been logarithmized. Each point in the figure represents a specific protein. The green points are downregulated proteins and red points represent upregulated proteins. **(B)**. GO enrichment analysis of SMS and SMN. The X-axis is the rich factor. The Y-axis is -log10 (*value of p*). Each bubble in the figure represents a secondary GO category. The size of the bubble is directly proportional to the number of concentrated proteins enriched in this GO secondary classification (BP, biological process; CC, cellular component; and MF, molecular function). **(C)**. KEGG enrichment analysis of DEPs between SMS and SMN.

The accession number of the proteomic data for SMS and SMN is PXD025290. The proteomic data showed 235 DEPs between SMS and SMN, among which 118 proteins were upregulated and 117 proteins were downregulated (Figure 10A). Subsequently, the DEPs were mapped to GO pathways and KEGG pathways.

According to the results of the GO functional analysis, it was clear that almost all the DEPs were enriched in biological processes and cellular components (Figure 10B). According to the results of the KEGG pathway analysis, 49 KEGG pathways of 109 DEPs were statistically significantly different between SMS and SMN. Further study indicated that the DEPs were involved in functions such as pilus (*p*=0.00001), pilus organizations (*p*=0.00012), cell adhesion (*p*=0.00006), protein secretion by the type II secretion system (*p*=0.00029), and protein transport across the cell outer membrane (*p*=0.00029), all of which were significantly upregulated in SMS compared with SMN (Figure 10C).

### Integration of Transcriptomic and Proteomic Analyses

**Figure 11:**
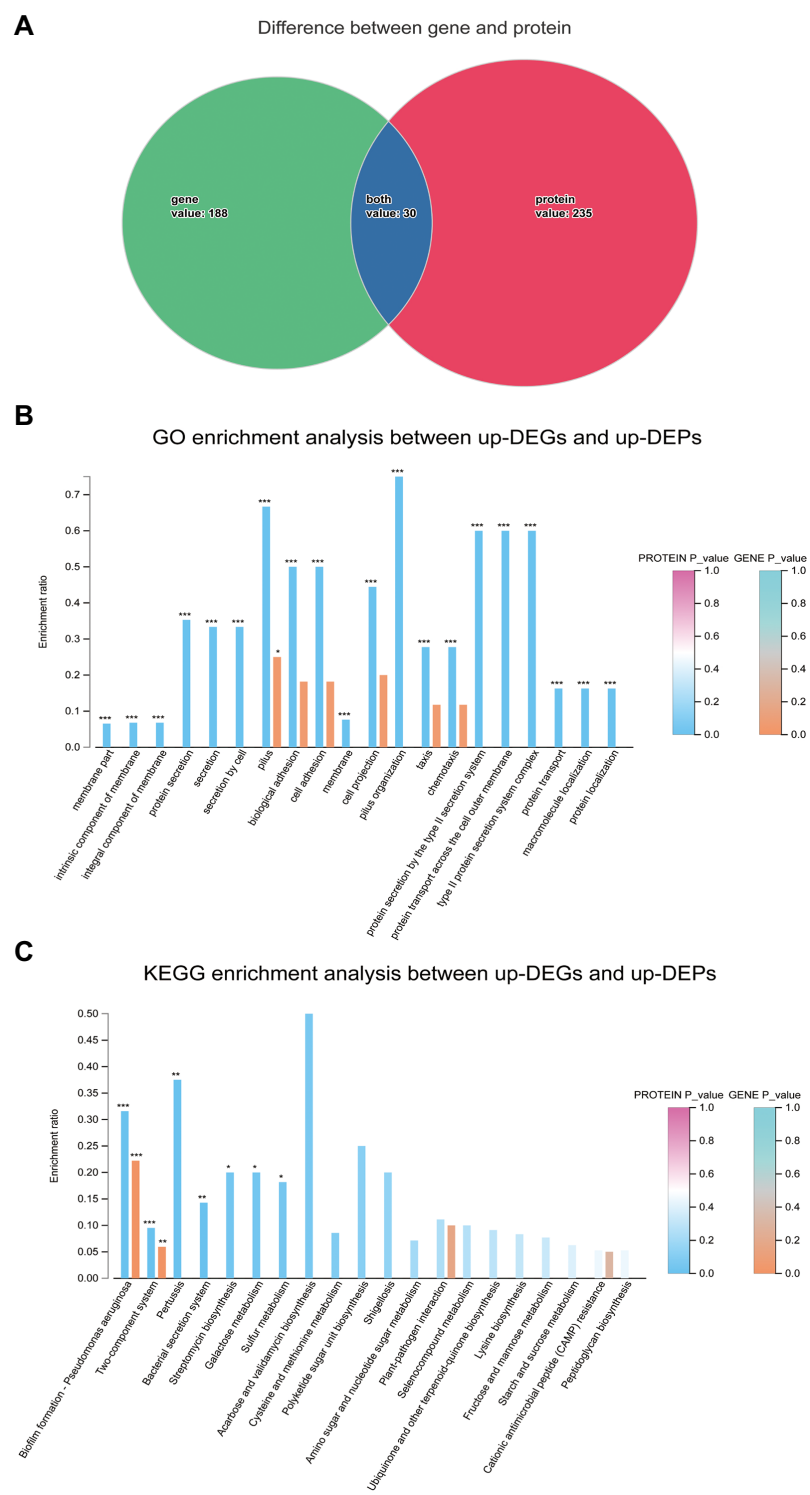
Integration of transcriptomic and proteomic analysis. **(A)**. The overlapping genes expressed differently in both the transcriptome and proteome. **(B)**. GO enrichment analysis between up-DEGs and up-DEPs. **(C)**. KEGG enrichment analysis between up-DEGs and up-DEPs.

To gain an understanding of the effects of SMG on *S. maltophilia* from a systematic biological perspective, the DEGs and DEPs were integrated to identify overlapping genes that were differentially expressed in both the transcriptome and proteome. In total, 30 genes were selected (Figure 11A), and genes with either upregulated or downregulated expression at both the mRNA level and protein levels were used for bioinformatic analysis. The GO enrichment analysis indicated that biological processes such as biological adhesion, cell adhesion, cell projection, and pilus organization might be affected in SMS. In addition, cellular components such as intrinsic components of the membrane, integral components of the membrane, membrane parts, and external encapsulating structure parts might also be affected in SMS (Figure 11B). The KEGG enrichment analysis indicated that biofilm formation and the two-component system between up-DEGs and up-DEPs might be affected in SMS (Figure 11C), which was in agreement with the DEGs enriched in the KEGG pathways at the transcriptomic level. Besides, four genes (chpC, pilJ, pilH, and pilG) involved in biofilm formation and seven genes (rpoN, chpB, chpC, pilA, pilG, pilH, and pilJ) involved in the two-component system were significantly upregulated.

## Discussion

The physiological changes and metabolic responses of microorganisms to the extreme environments of space have attracted increasing attention. Monitoring microorganisms in space stations plays an important role in monitoring the safety of spacecraft and the health of astronauts. With the advancement of science and technology, more space and ground-assisted research technologies have helped us better understand the impact of the space environment on microorganisms. In recent years, a large number of studies have reported on the effects of space environment and simulated space environment on the physiological characteristics of microorganisms and the relationship between microorganisms and human beings. However, *S. maltophilia*, as a new opportunistic pathogen that is found in water, soil, humans, and animals, has rarely been studied for its effect on the space environment. *Stenotrophomonas maltophilia* is an important nosocomial pathogen and is closely related to clinical infections, especially in immunocompromised people. Hence, astronauts with low immunity are susceptible to *S. maltophilia* during spaceflight. Currently, there is no integrated genetic information available for *S. maltophilia* after cultivation in the SMG environment, which makes it difficult to explore the physiological changes and metabolic responses of the organism after exposure to this unique condition. Overall, our results confirm that in SMG environment, the physiological characteristics of *S. maltophilia* have undergo some changes, such as a faster growth rate, enhanced biofilm formation ability, and increased mobility. Furthermore, through multiomics analysis, 118 DEPs were found to be upregulated, most of which were related to biofilm formation, pilus organization, bacterial motility, locomotion, and the bacterial secretion system. At the genetic level, 113 DEGs were upregulated. For instance, DEGs such as pilP, pilM, flgE, flgG, and ronN, which are related to cell motility, were significantly upregulated. In addition, the chpB, chpC, rpoN, pilA, pilG, pilH, and pilJ genes, which are related to biofilm formation and cell outer membrane, were also significantly upregulated. These upregulated DEGs were consistent with the upregulated DEPs.

Considering the different culture conditions, cultivation times, and strains used, previous study showed that microorganisms exposed to a space environment or cultivated in the SMG environment on the ground exhibited different growth characteristics. Most researchers have shown that microgravity or weightlessness in space can significantly increase the growth rate of bacteria (Benoit and Klaus, 2007; Kim et al., 2013; Javadi et al., 2014; Yim et al., 2020). Compared with ground group, some studies have shown that under microgravity or weightlessness conditions, the growth rate of some bacteria does not change significantly (Coil et al., 2016; Fajardo-Cavazos et al., 2018). A few studies have even shown that the flight strain has a significantly decreased growth rate compared with that the normal gravity group (Fajardo-Cavazos and Nicholson, 2016). Our study illustrated (Fajardo-Cavazos and Nicholson, 2016) that SMS exhibited an increased growth rate compared with that of SMN and SMO. It is worth noting that under SMG or weightlessness, the underlying mechanism of the microbial growth rate is still unclear. Some experts believe that this may be related to the increase in the utilization of nutrients by bacteria in a specific environment, and the corresponding reduction in extracellular material transport (Brown et al., 2002). Through the GO enrichment analysis performed in this research, we found that the increased growth rate of SMS might be related to some upregulated DEPs involved in biological regulation, metabolic process, cellular anatomical entity, and catalytic activity. Moreover, according to the KEGG pathway analysis, some of the upregulated DEPs in amino acid metabolism, energy metabolism, and carbohydrate metabolism were also central to the increased growth rate. In addition, according to the COG annotations, 20 DEPs related to intracellular trafficking, secretion, and vesicular transport were overexpressed in SMS. In conclusion, we believe that the accumulation of these changes may increase the growth rate of SMS.

Astronauts during spaceflight are in a state of reduced immunity, which accompanied by changes in microbiota. Some studies have found that some characteristics of bacteria have a certain correlation with their motility (Portela et al., 2019). The differences in the structure and number of flagella, fimbriae, and pili of bacteria greatly affect their motility. As a key microbial behavior, motility plays a key role in nutrient absorption, tissue localization and invasion, biofilm formation, and pathogenicity. Changes in motility may cause changes in the bacterial distribution in the human body, and these motility changes may damage the health of astronauts. Therefore, the effect of microgravity on bacteria can be observed and explained by the change in bacterial motility to a certain extent. Although, domestic and foreign researchers have studied the influence of microgravity on microbial motility, the exact way in which microgravity causes these changes is still unclear. A reasonable explanation suggests that bacteria are affected by microgravity on account of the quiescent fluid environment enclosing bacteria in a liquid suspension culture, and another explanation is that the buoyant convection of low dense fluid in the vicinity of cell plays a key role (Tirumalai et al., 2017). Portela et al. (2019) found that after spaceflight, the morphology of *Escherichia coli* changed under Transmission Electron Microscope (TEM). Compared with the normal gravity group, the samples after spaceflight showed a decreased size and an increased number of outer membrane vesicles. Benoit and Klaus (2007) revealed that under the same spaceflight conditions, motile bacteria displayed greater cell numbers than nonmotile bacteria. Microbial motility is meditated by an electromotive gradient of ions across the cell membrane, so the disparity between each concentration and nutrient depletion produced by microgravity and SMG may have led to the difference in motility (DeRosier, 1998). Wilson et al. (2007) revealed that *Salmonella Typhimurium* cultivated in LB in HARVs showed a decrease in motility; downregulations of fliEST, flhD, and flgM; and upregulation of filC. Crabbé et al. (2011) found upregulated gene expression related to motility in *Pseudomonas aeruginosa* under SMG, including fliACDGS, fleLNP, and flgM, but there were no obvious changes in the expression of genes related to motility after spaceflight. Duscher et al. (2018) revealed that *Vibrio fischeri* showed a trend of upregulation of flhA and flgM after 12 and 24h of cultivating in SMG. Interestingly, Tucker et al. (2007) found that even in the same HARV environment, *E. coli* in different nutrients displayed different motility and had different expression of genes related to motility. In this study, compared with SMN and SMO, SMS showed more bacterial intercellular mucus and rougher membranes under 20,000x SEM. The swimming experiment revealed that the bacteria in the SMS group had increased motility compared with those in the SMN and SMO groups. In addition, considering that the swimming experiment was conducted under ground conditions and there would be some variables affecting the results, we will take videos of precultured bacterial cells directly from three samples to compare their motility in the subsequent experiment. Through the GO enrichment analysis performed in this research, we found that the increased motility of the SMS group may be related to some upregulated DEPs involved in locomotion, localization, biological adhesion, and binding. Moreover, according to the COG annotations, ten DEPs related to cell motility and seven DEPs related to cell wall, membrane, and envelope biogenesis were overexpressed in SMS. In addition, according to the GO enrichment analysis and KEGG enrichment analysis, DEGs, including pilP, pilM, flgE, flgG, and ronN, which are associated with cell motility, were overexpressed in SMS. These upregulated DEGs were consistent with the changes in the DEPs. In conclusion, we believe that the overlap of these changes may increase the motility of SMS in the SMG environment.

Most of the studies on *S. maltophilia* biofilms are related to the effects of antibiotics, hormones, or combinations of drugs. For instance, Wang et al. (2016a) discovered the synergistic inhibition of fluoroquinolones and azithromycin on the biofilm formation of *S. maltophilia*, and Ciacci et al. (2019) found an *in vitro* synergistic therapeutic effect of colistin and N-acetylcysteine on *S. maltophilia*. However, there are few studies on the characteristics of *S. maltophilia* in the space environment and SMG. Therefore, we focused on microgravity as a starting point to investigate the physiological characteristics of *S. maltophilia* in a microgravity environment. The formation of biofilms in bacteria was first carried out under microgravity in 2001 (McLean et al., 2001). After 24h of exposure to microgravity, the formation of biofilms in *E. coli* increased (Lynch et al., 2006). *Escherichia coli* were cultured in a rotary cell culture system under microgravity and the biofilm formation ability increased in the SMG group compared with that in the NG group (Yim et al., 2020). The biofilm formation of the bacterial pathogen *S. typhimurium* was also increased after the space shuttle mission STS-115 (Wilson et al., 2007). These results were consistent with our results. However, Zhao et al. (2019) found that *Acinetobacter baumannii* showed decreased biofilm formation after a spaceflight on the Shenzhou 11 spacecraft of China. *Klebsiella pneumoniae* formed different subgroups, M1 and M2, on solid medium, and an increased biofilm forming ability was found in M1 compared with that in M2 in SMG (Wang et al., 2017). Therefore, there is no general conclusion on the influence of microgravity on microorganisms. The differences in strains, culture methods, and durations spent in microgravity may lead to different results. In our study, the biofilms that formed at the bottom of the HARV bioreactors in the SMS group were thicker than those in the SMN group. Quantitative biofilm experiments showed that the biofilm formation ability of SMS was more than twice that of either SMN or SMO. CLSM analysis illustrated that the biofilm thickness in SMS was thicker than that in SMN or SMO. Through the GO and KEGG enrichment analyses performed in this research, we found that the biofilm formation ability of SMS might be related to some of the upregulated DEPs involved in intrinsic components of the membrane, integral components of the membrane, type II protein secretion systems, galactose metabolism, and sulfur metabolism. Moreover, according to the COG annotations, seven DEPs related to cell wall, membrane, and envelope biogenesis and 20 DEPs related to intracellular trafficking, secretion, and vesicular transport were overexpressed in SMS. In addition, according to the GO enrichment analysis and KEGG enrichment analysis, DEGs, including the chpB, chpC, rpoN, pilA, pilG, pilH, and pilJ genes, which are associated with biofilm formation and cell outer membrane, were overexpressed in SMS. These upregulated DEGs were consistent with the changes in the DEPs. In conclusion, we believe that these changes may increase the biofilm formation of SMS in the SMG environment.

To sum up, the changes in genome, transcriptome, and proteome can to some extent explain the phenotypic changes such as motility and biofilm formation ability. The present study might serve a basis for future studies of the complex mechanism by which bacteria adapt to the space. However, the overall changes in *S. maltophilia* at the level of DNA or RNA are still limited. Considering the complexity of space environment and the limitation of simulated experiment condition on the ground, more relevant experiments are needed for deep research.

## Data Availability Statement

All the data used to draw conclusions are presented in the article. The sequence reads used for WGS and RNA-seq have been submitted to NCBI with the project accession numbers of PRJNA720482 and PRJNA720572. The mass spectrometry proteomics data have been deposited to the ProteomeXchange Consortium *via* the PRIDE (Deutsch et al., 2020) partner repository with the dataset identifier PXD025290.

## Author Contributions

CL and JW designed and coordinated the project. XS performed the laboratory experiments and wrote the manuscript with assistance from all the authors. YG, TF, XJ, DW, DL, PB, and BZ performed the data analysis. All authors contributed to the article and approved the submitted version.

## Conflict of Interest

The authors declare that the research was conducted in the absence of any commercial or financial relationships that could be construed as a potential conflict of interest.

## Publisher’s Note

All claims expressed in this article are solely those of the authors and do not necessarily represent those of their affiliated organizations, or those of the publisher, the editors and the reviewers. Any product that may be evaluated in this article, or claim that may be made by its manufacturer, is not guaranteed or endorsed by the publisher.
