# Supplementary figures and images for: Effects of Simulated Microgravity on the Physiology of Stenotrophomonas maltophilia and Multiomic Analysis
Source: Front Microbiol. 2021 Aug 27;12:701265. doi: 10.3389/fmicb.2021.701265 (PMC8429793; doi:10.3389/fmicb.2021.701265)

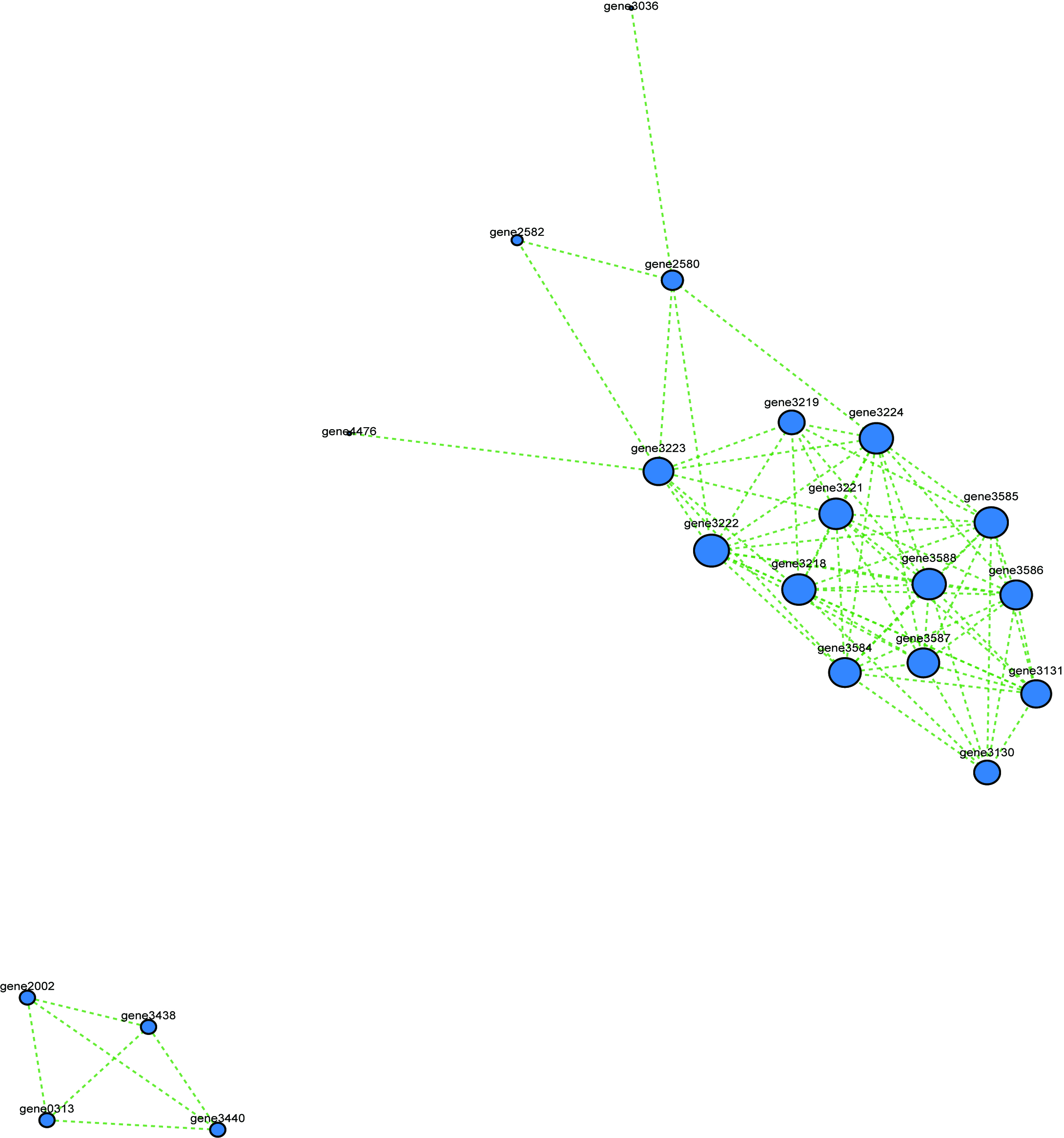

Supplement: Supplementary file 1 [file Data_Sheet_1.ZIP › PPI network diagram of associated data.jpg]
